# NEK2 promotes esophageal squamous cell carcinoma cell proliferation, migration and invasion through the Wnt/β-catenin signaling pathway

**DOI:** 10.1007/s12672-023-00692-5

**Published:** 2023-05-26

**Authors:** Dong Guo, Weinan Yao, Xingyu Du, Jing Dong, Xueyuan Zhang, Wenbin Shen, Shuchai Zhu

**Affiliations:** Department of Radiation Oncology, Fourth Hospital of Hebei Medical University, Shijiazhuang, 050000 China

**Keywords:** NEK2, Invasion, Wnt/β-catenin, Esophageal squamous cell carcinoma, Proliferation

## Abstract

**Objectives:**

The NEK2 (never in mitosis gene A-related kinase 2), a serine/threonine kinase involved in chromosome instability and tumorigenesis. Hence, this study aimed to explore the molecular function of NEK2 in esophageal squamous cell carcinoma (ESCC).

**Methods:**

By available transcriptome datasets (GSE53625 cohort, GSE38129 cohort, and GSE21293 cohort), we analyzed the differentially expressed genes in invading and non-invading ESCC. Subsequently, we evaluated the association between NEK2 expression level and clinical outcomes through Kaplan–Meier analysis method. The quantitative real-time polymerase chain reaction (qRT-PCR) and western blotting (WB) analyses were performed to determine the expression levels of NEK2 mRNA and protein, respectively. We knocked down the NEK2 expression in ESCC cells (ECA109 and TE1), and evaluated the NEK2 biology function associated with ESCC cell proliferation, migration, invasion, and colony formation abilities. Finally, the downstream pathway of NEK2 was analyzed through Gene Set Enrichment Analysis (GSEA) and validated the regulatory mechanism of NEK2 on the potential pathway through WB.

**Results:**

We found that NEK2 was highly expressed in ESCC cells compared with human esophageal epithelial cells (HEEC) (P < 0.0001), and high NEK2 expression was remarkably associated with poor survival (P = 0.019). Knockdown of NEK2 showed the significant inhibitory effect for tumorigenesis, and suppressed the ESCC cells proliferation, migration, invasion, and formation of colonies abilities. Additionally, GSEA revealed that Wnt/β-catenin pathway was a downstream pathway of NEK2. WB results further validated the regulatory mechanism of NEK2 for Wnt/β-catenin signaling.

**Conclusions:**

Our results indicated that NEK2 promotes ESCC cell proliferation, migration and invasion by activating the Wnt/β-catenin pathway. NEK2 could be a promising target for ESCC.

## Introduction

Esophageal cancer (ESCA) is a common digestive system malignancy and a major global public health problem. In recent years, the incidence of ESCA has gradually increased, and the incidence of ESCA in men was higher than women [1, 2]. In China, esophageal squamous cell carcinoma (ESCC) is the most prevalent histologic type of ESCA [3]. The potential factors contributing to the ESCC may be smoking, alcohol abuse, drinking hot water, eating too-hot food and other bad habits [4]. Most ESCC patients are diagnosed with an advanced-stage on account of the lack of significant clinical symptoms, and these patients usually develop lymph node metastasis resulting in poor clinical outcomes [5]. Despite obtaining the great advances of diagnosis and therapeutic methods for ESCC, the response for ESCC is unsatisfactory [6]. Hence, it is indispensable to reveal the molecular mechanism of ESCC progression and identify the trustworthy therapeutic targets for ESCC.

The never in mitosis gene A (NIMA)-related kinase 2 (NEK2) is a serine/ threonine kinase that belongs to the NIMA-related kinase family, which participates in the multiple biological regulatory processes, including microtubule stabilization, centrosome separation, cell cycle control and DNA damage response [7–9]. Increasing evidence has demonstrated that NEK2 is over-expressed in cervical cancer [10], breast cancer [11], head and neck squamous cell carcinoma [12], and colorectal cancer [13]. Additionally, the biological role of NEK2 in tumorigenesis has been preliminarily studied. NEK2 is regarded as a trustworthy target in cancer treatment, which is linked to tumorigenesis, migration, invasion and survival [14–16]. NEK overexpression, for example, has been demonstrated to promote gastrointestinal cancer proliferation through the regulation of the cell cycle and apoptosis [17]. Hayward et al. showed that overexpression NEK2 was associated with poor survival outcomes in colorectal cancer [18]. Notably, a recent study showed that knockout of NEK2 expression suppressed breast lung cancer cells migration and invasion by mutant EGFR, thereby exerting anti-tumor effect [19]. These findings suggest that targeting NEK2 may enhance the treatment efficacy, whereas the precise regulatory mechanism of NEK2 for oncogenesis in ESCC remains to be clarified.

The Wnt signaling has been demonstrated that releases a vital role in multiple biological signal transduction process, which is triggered by specific signaling protein and delivers the signals to targeted cells [20, 21]. The activation of Wnt signaling can facilitate the tumorigenesis, and Wnt pathway aberrant alterations result in occurrence of lung cancer, colorectal cancer, nasopharyngeal carcinoma, cervical cancer [22–25]. Additionally, β-catenin serves as a crucial activator of Wnt signaling pathway, and the dephosphorylated of β-catenin further contributes the epithelial–mesenchymal transformation (EMT) [26]. The growing evidence studies have revealed that the activation of the Wnt/β-catenin signaling pathway is significantly associated with EMT [27]. As a member of a NIMA-related family proteins, we hypothesize that NEK2 may facilitate the EMT by regulating the Wnt/β-catenin pathway and thereby results in accelerating deterioration of ESCC.

In this study, we analyzed the expression level of the NEK2 in ESCC cells and human esophageal epithelial cells (HEEC), and demonstrated that NEK2 overexpressed in ESCC cells. Knockdown of NEK2 inhibited the proliferation, migration, and invasion in ESCC cells. Notably, NEK2 could regulate Wnt/β-catenin signaling pathway to promote tumorigenesis of ESCC.

## Materials and methods

### Bioinformatic analysis

The mRNA expression data of ESCC tissues and normal esophageal tissues in the Gene Expression Omnibus (GEO) were obtained, and the differentially expressed genes data of GSE53625 cohort (179 ESCC tissues and 179 adjacent tissues), GSE38129 cohort (30 ESCC tissues and 30 adjacent tissues) were analyzed with the specific filter criteria (|LogFC| ≥ 1, adjusted P < 0.05). Additionally, the differentially expressed genes between 12 invading ESCC tissues and 23 non-invading ESCC tissues were identified in GSE21293 cohort, and the ROC curve was draw to exhibit the predictive ability of NEK2 for invasion. Kaplan–Meier (KM) survival curves were depicted using the “survminer” and “survival” packages. Subsequently, we explored the vital signal pathway modulated by NEK2 based on the NEK2 expression data in ESCC patients.

We used the CIBERSORT algorithm to obtain the normalized gene expression matrix and evaluated the infiltration levels of 22 immune cell infiltration. Finally, the correlations between NEK2 expression and infiltration levels of immune cells, immune checkpoints were analyzed through Spearman’s correlations.

### Cell lines and culture

Human esophageal epithelial cells (HEEC) and ESCC cell lines (ECA109, TE1, KYSE410 and KYSE510), obtained from the Scientific Research Center of the fourth Hospital of Hebei Medical University (Shijiahzuang, China), were routinely maintained in the DMEM supplemented with 10% fetal bovine serum (FBS) and RPMI-1640 medium containing 10% FBS, respectively. These cells were cultured in a humidified incubator at 37 °C containing 5% CO_2_.

### Cell transfection

ECA109 and TE1 cells were seeded and cultured in 6-well plates. These cells were transfected when cells reached 50–60% density. To establish transfection compound with target gene knockdown, we purchased NEK2-targeted small interfering RNAs (si-NEK2) from RIBOBIO (Guangzhou, China). The si-NEK2 was transfected into these cells using Lipofectamine 2000 (Invitrogen, USA). The siRNAs sequences are shown in Table 1. After 24–48 h of cells transfection, the knockdown efficiency was verified through qRT- PCR and western blotting (WB) assays.

**Table 1:** The sequences of siRNAs

Name	Sequence
si-NC	TTCTCCGAACGTGTCACGT
si-NEK2-1	CCAGCCCTGTATTGAGTGA
si-NEK2-3	GTCCTACAATGAGAAATCA

### Quantitative real-time polymerase chain reaction assay (qRT-PCR)

Total RNA from cultured cells was extracted using TRIzol reagent (Invitrogen,USA) according to the manufacturer’s instructions, and corresponding cDNA was generated using a RevertAid RT Reverse Transcription Kit (Thermo Fisher Scientific, USA) with specific conditions ( 42 °C with 60 min and 70 °C with 5 min). qRT-PCR assay was conducted using MonAmp™ SYBR^®^ Green qPCR Mix (Monad Biotech Co., Ltd.) based on the manufacturer’s instructions. The RNA results were standardized based on the GAPDH levels, and relative level of NEK2 wad calculated by the 2^−ΔΔCt^ method [28]. The sequences of NEK2 and GAPDH were designed and applied (NEK2 forward: 5′-CGACGGTTAAACGGGGC-3′; NEK2 reverse: 5′-TACAGCAAGCAGCCCAATGA-3′; GAPDH forward: 5′-CGCTGAGTACGTCGTGGAGTC-3′; GAPDH reverse: 5′-GCTGATGATCTTGAGGCTGTTGTC-3′).

### Protein extraction and WB assays

Total protein extraction of cell lines was conducted using radio immunoprecipitation assay (RIPA) buffer and phosphatase inhibitors (Roche, Sigma, St. Louis MO, USA). Subsequently, we used the BCA protein concentration determination kit (Beyotime Institute of Biotechnology) to quantitate the protein concentration. Extracted proteins were resolved by SDS-PAGE and transferred to 0.22 μm PVDF membranes. The membranes were incubated with the corresponding primary antibody at 4 °C for 16 h, followed by secondary antibody with 1 h at room temperature. Finally, the protein bands were presented using the Odyssey Infrared Imaging System (LI-COR Biosciences, Lincoln, NE, USA). The relevant antibodies used for this study were antiNEK2 (sc55601, Santa Cruz Biotechnology), anti-E-cadherin (60335-1-Ig, Proteintech), anti-Vimentin (10366-1-AP, Proteintech), anti-N-cadherin (22018-1-AP, Proteintech), anti-MMP2 (ab97779, Abcam), anti-MMP9 (ab76003, Abcam), β-catenin (ab32572, Abcam), anti-cyclin D1 (1:200 dilution, ab16663, Abcam), anti- c-Myc (ab32072, Abcam), anti-GAPDH (60004-1-Ig, Proteintech).

### Cell viability and colony formation

The ECA109 and TE1 cells with knockdown of NEK2 were seeded into 96-well plates (4.0 × 10^3^ cells per well) for cell counting kit-8 (CCK8) assay (Med Chem Express (MCE) Princeton, NJ, USA). After 24, 48, 72 and 96 h, 10 µL CCK8 solution was added to each well, and absorbance of each well was measured for cells viability at 450 nm after 2 h. For clonogenic assays, 1.0 × 10^3^ ECA109 and TE1 cells/well were cultured in 6-well plates. After 14 days of growth, these cells were fixed in paraformaldehyde (4%) for 15 min, and stained with crystal violet (0.1%) for 10 min. Cell clones (> 50 cells) were counted and compared using a microscope (Olympus, Japan).

### Cell migration and invasion

The cell migration ability was tested through wound-healing assay. Briefly, cells were cultured in a 6-well plate and scratched for a straight-line with a 200 uL pipette tip when the cell density reached 80%. The ECA109 and TE1 cells were cultured in a serum-free medium, and microscope was applied to photograph and calculate the wound healing between scratches in different groups in different periods (0 h, 24 and 48 h). 24-well transwell chamber (BD Biosciences) and Matrigel (Corning Inc., Corning, NY, USA) were used to evaluate the cell invasive ability. Cells (1.0 × 10^5^cells per chamber) were seeded to the upper chamber, and 20% FBS medium was added into lower chamber as a chemoattractant. 24 h of incubation later, cells pass through the Matrigel and entered the lower chamber, of which were fixed and stained with 0.1% crystal violet. The cells that stained blue were visualized by a fluorescence microscope (Nikon Ti2, Japan), and then photographed and counted in five random fields for further statistical analysis.

### Statistical analysis

Statistical analysis was performed using Graphpad Prism version 8.0. Student’s t-test was used for comparison between two groups, and one-way analysis of variance (ANOVA) was applied to evaluate the differences among multiple groups. Receiver operating characteristic (ROC) curves analysis was conducted to evaluate the predictive ability of NEK2 levels for invasion status in ESCC patients. Kaplan–Meier survival curves was generated with a log-rank test. The values of P < 0.05 with two sides indicate a significant statistical difference.

## Results

### Molecular characteristics of NEK2 in transcriptome cohorts

**Fig. 1:**
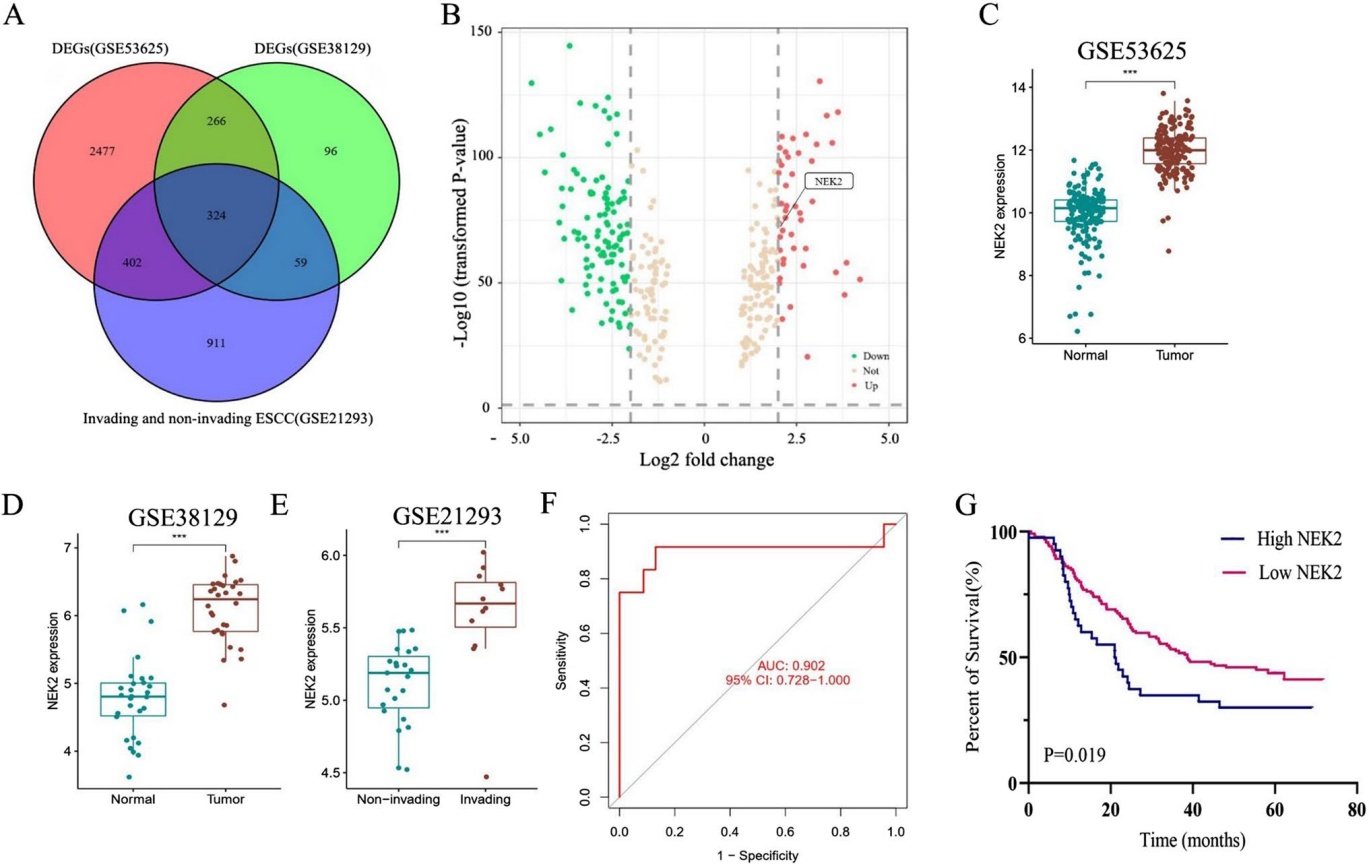
NEK2 is an oncogene associated with ESCC. **A** Venn plots of overlapping DEGs among GSE53625, GSE38129 and GSE21193 cohorts. **B** Volcano map of DEGs. **C**–**E** Differential expression levels of NEK2 in ESCC. **F** ROC analysis of the NEK2 expression in predicting the invading ESCC patients. **G** Prognostic significance of NEK2 in patients with ESCC by the KM plotter method. *DEGs* differentially expressed genes, *ESCC* esophageal squamous cell carcinoma; *P < 0.05, **P < 0.005, ***P < 0.001

After mRNA expression data standardizing and processing, 3469 differentially expressed genes in the GSE53625 and 745 differentially expressed genes in GSE38129 were obtained. We then intersected these differentially expressed genes with the list of 1696 invasion related genes (GSE21293) as shown in the Venn diagrams in Fig. 1A. Among them, NEK2 is overexpressed and facilitates oncogenesis in malignancies. Analysis of GSE53625 cohort and GSE38129 cohort data indicated that the NEK2 expression level in ESCC tissues was upregulated compared with normal tissues (Fig. 1B–D). Next, GSE21293 database was used to further analyze the expression of NEK2 between invading ESCC and non-invading ESCC. As shown in Fig. 1E, compared to non-invading ESCC, the NEK2 expression had remarkably higher expression level in invading ESCC (P < 0.001). Figure 1F depicted the ROC curves, the results illustrated the reliable predictive ability of NEK2 for invasion status in GSE21293 cohort, with a relatively high sensitivity (AUC = 0.902). Of note, the survival analyses indicated the patients with high NEK2 expression had poor clinical outcomes than patients with low NEK2 expression in GSE53625 cohort (P = 0.019, Fig. 1G). The above findings suggested that the NEK2 expression between ESCC tissues and normal tissues generated alterations, speculating a crucial regulatory mechanism for NEK2 in the tumorigenesis of ESCC.

### The correlation of NEK2 expression with immunological characteristics in ESCC

**Fig. 2:**
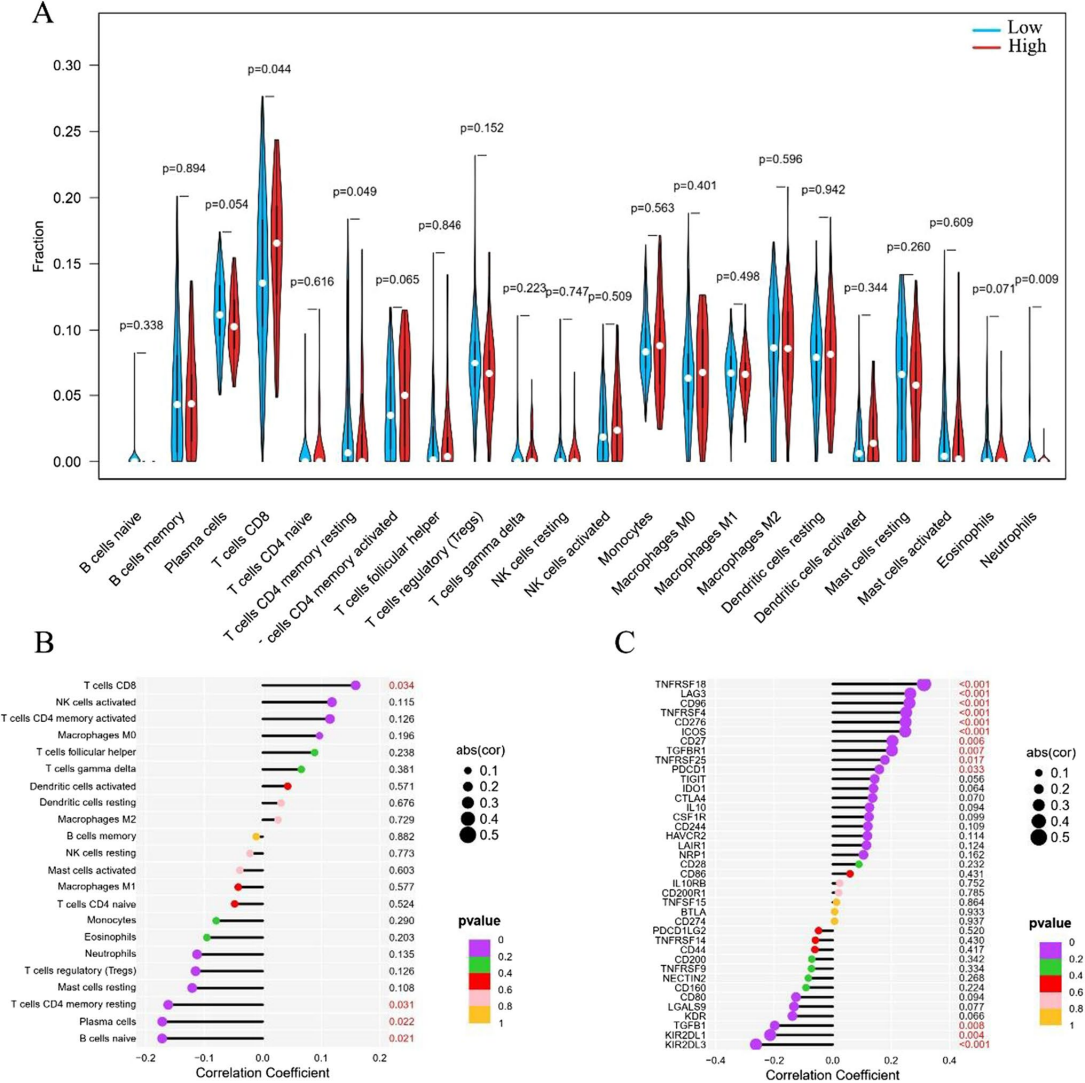
Evaluation of immunological characteristics in patients with ESCC. **A** The infiltration proportion of immune cells in the high and low NEK2 groups in ESCC. **B**, **C** The correlation of NEK2 with infiltration level of immune cells and immune checkpoint in ESCC. *ESCC* esophageal squamous cell carcinoma

**Fig. 3:**
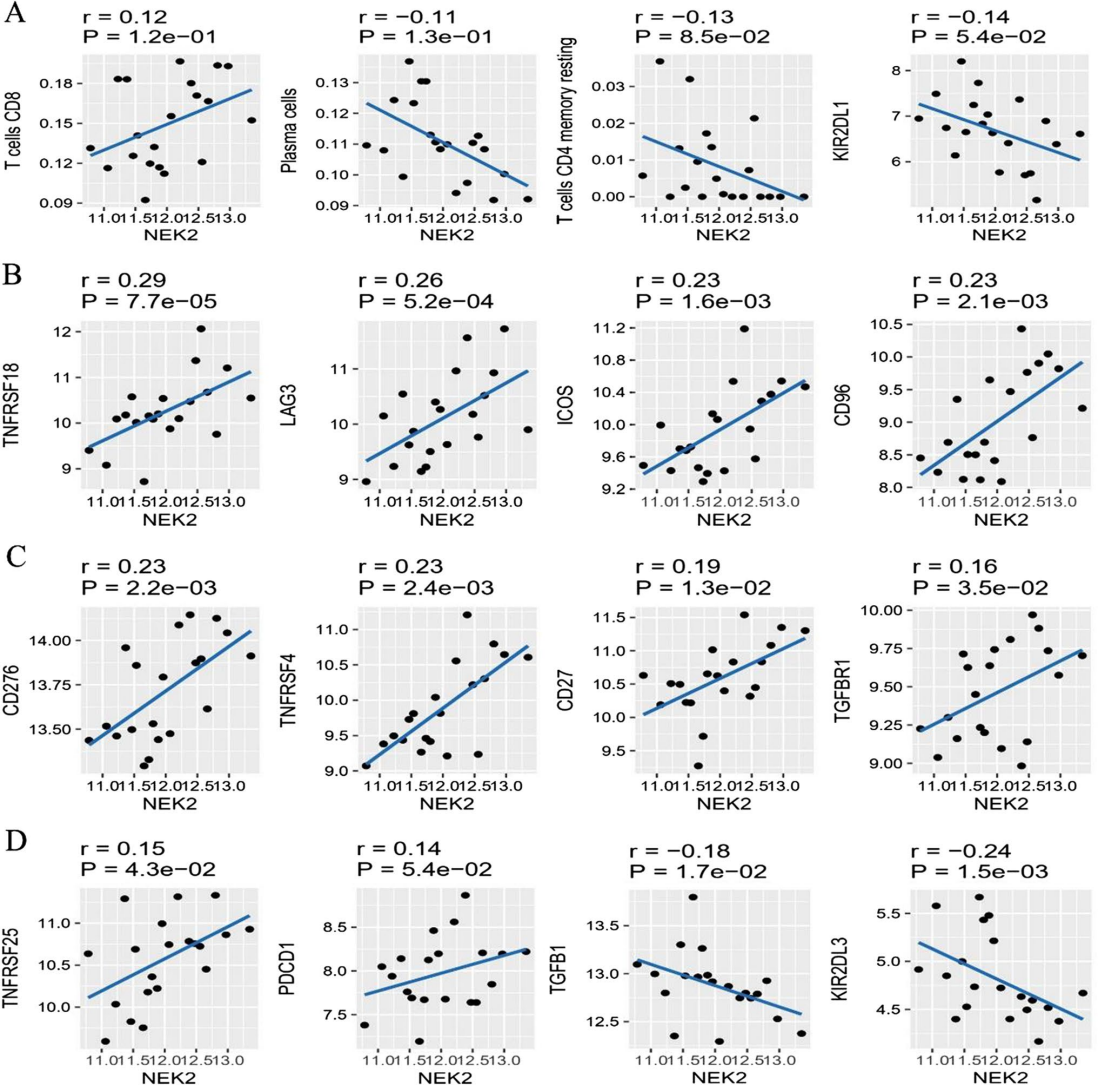
NEK2 correlated with the immune cell infiltration and immune checkpoint-related genes in ESCC. **A** The Spearman’s correlations of NEK2 with infiltration level of immune cells. **B**–**D** The Spearman’s correlations of NEK2 with immune checkpoint-related genes. *ESCC* esophageal squamous cell carcinoma

To further analyze the relationship between the NEK2 expression and immune cell infiltration, we evaluated the fraction of immune cell infiltration after integrating NEK2 gene expression (Fig. 2A). The dysregulated levels of infiltrating immune cells and different immune checkpoints expression are shown in Fig. 2B, C, respectively. Spearman’s correlations analyses demonstrated that NEK2 expression was positively associated with the enrichment of T cell CD8 while negatively associated with Plasma cells and T cell CD4 memory resting. Additionally, we found that NEK2 expression was positively associated with many immune checkpoints, including TNFRSF18, LAG3, ICOS, CD96, CD276, TNFRSF4, CD27, TGFBR1, TNFRSF25 and PDCD1 (Fig. 3). To sum up, we concluded that NEK2 participated in the immunity regulation in ESCC.

### NEK2 expression was upregulated in ESCC cell lines

**Fig. 4:**
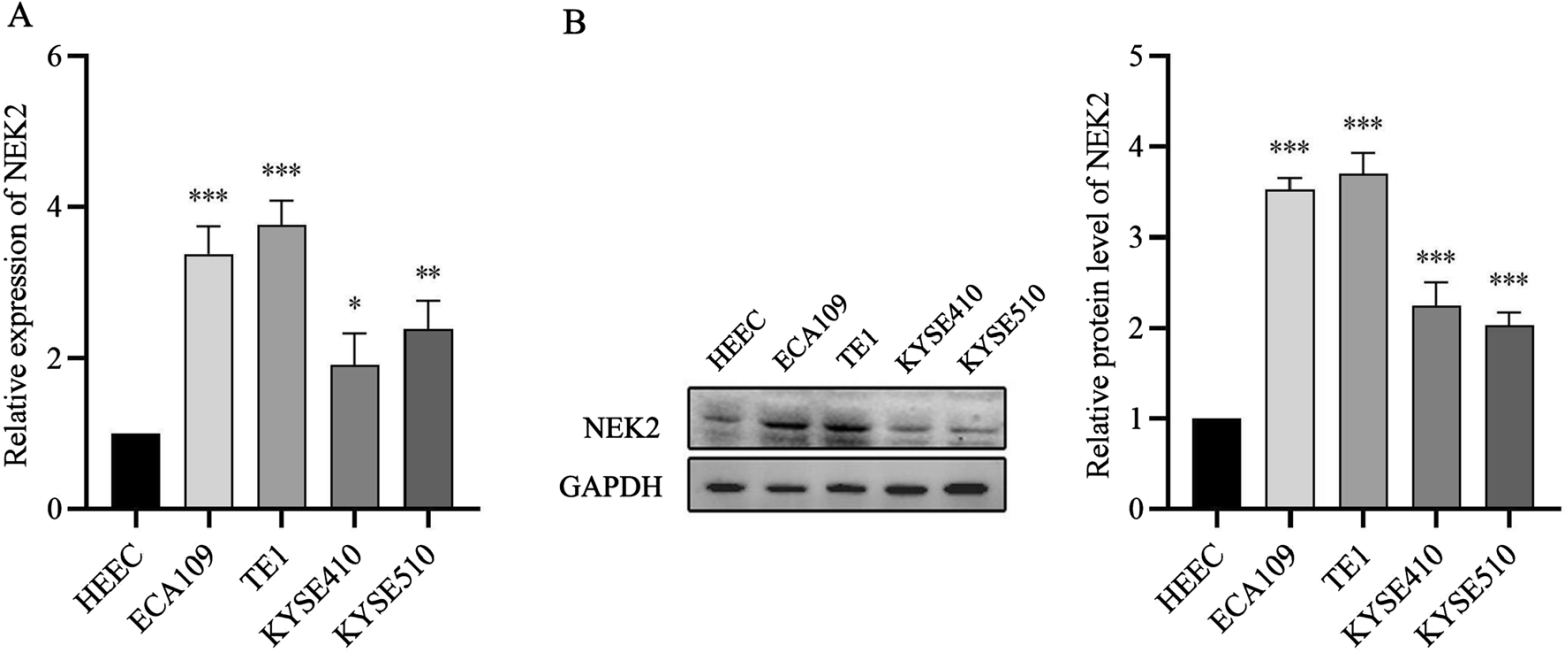
High NEK2 expression in ESCC cells. **A** qRT-PCR measurement of NEK2 expression in human esophageal epithelial cells (HEEC) and ESCC cell lines (ECA109, TE1, KYSE410 and KYSE510). **B** Western blot analyses of NEK2 protein expression in esophageal cells. *ESCC* esophageal squamous cell carcinoma; *P < 0.05, **P < 0.005, ***P < 0.001

To validate the reliability of public database transcriptome data associated with ESCC, the NEK2 expression in HEEC, ECA109, TE1, KYSE410 and KYSE510 were analyzed by qRT-PCR (Fig. 4A) and WB assays (Fig. 4B). The results showed that the mRNA (P < 0.05) and protein (P < 0.05) expression levels of NEK2 in ECA109, TE1, KYSE410 and KYSE510 cells were significantly upregulated that in HEEC cells.

### Knockdown of NEK2 inhibited the proliferation of ESCC cells

**Fig. 5:**
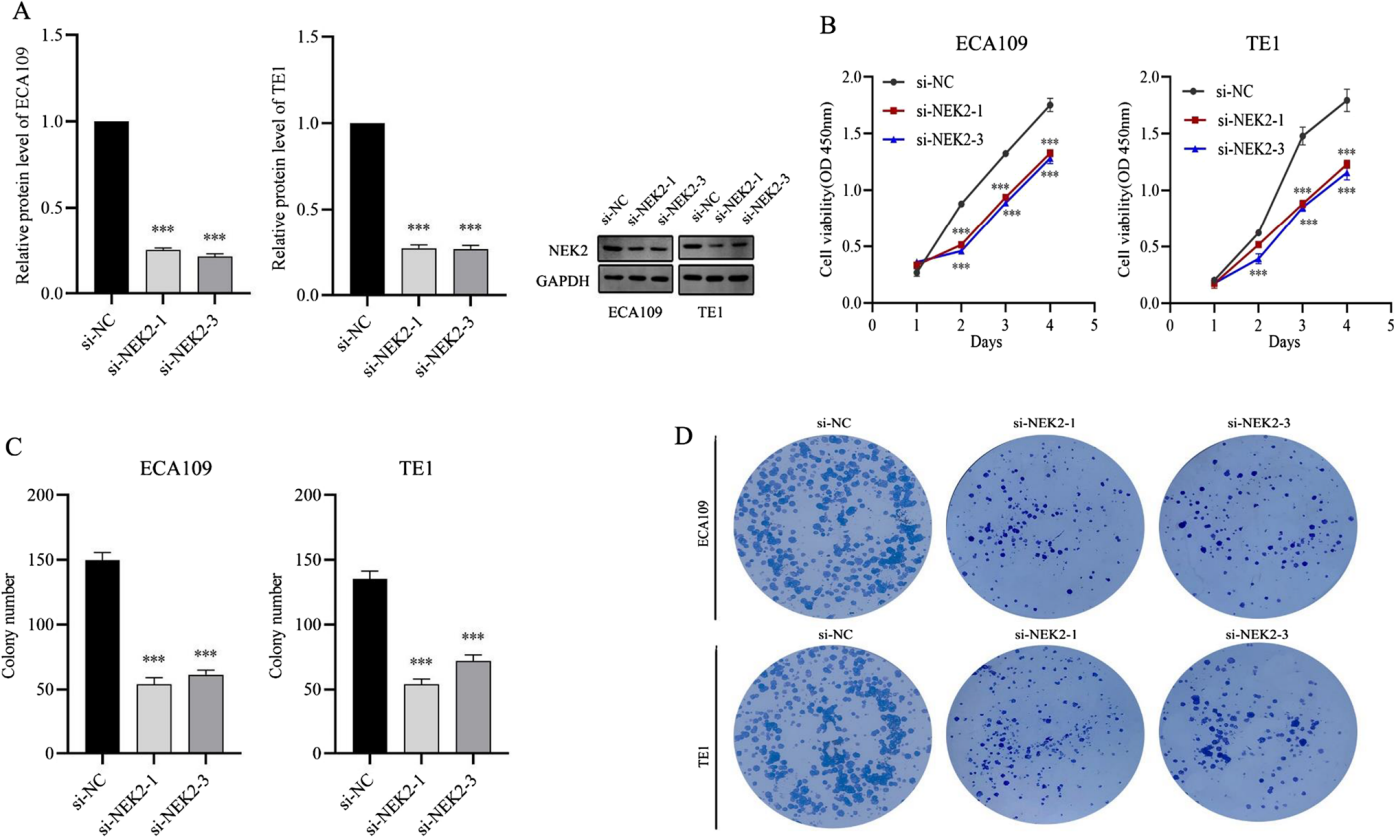
Knockdown of NEK2 inhibited proliferation of ESCC cells. **A** Western blot analyses of transfection efficiency with NEK2 siRNAs in ECA109 and TE1 cells. **B** CCK-8 assay showed knockdown of NEK2 inhibited cell viability in ECA109 and TE1 cells. **C**, **D** Effect of NEK2 knockdown on the colony formation capacity of ECA109 and TE1 cells. *ESCC* esophageal squamous cell carcinoma. *P < 0.05, **P < 0.005, ***P < 0.001

Through above mRNA and protein levels analysis with five different ESCC cell lines, we found NEK2 was relatively overexpressed in ECA109 and TE1 cell lines. Subsequently, we transiently transfected ECA109 and TE1 cells with si-NEK2 to construct the knockdown of NEK2 ESCC cell lines. We validated the knockdown efficiency in protein levels, and knockdown of NEK2 ECA109 and TE1 cells were applied for subsequent assays. The protein expression was significantly decreased after knockdown of NEK2 (Fig. 5A). CCK8 and colony formation assays were used to investigate the proliferation viability of ESCC cells. The CCK8 assay demonstrated that knockdown of NEK2 decreased the proliferation rate of ECA109 and TE1 cells (P < 0.05, Fig. 5B). The colony formation assay demonstrated that there was noteworthy inhibition of colony numbers in the NEK2 knockdown group when compared to the si-NC group (P < 0.05, Fig. 5C, D). This suggested that knockdown of NEK2 weakened ESCC cell proliferative ability.

### Knockdown of NEK2 attenuated migration and invasion of ESCC cells

**Fig. 6:**
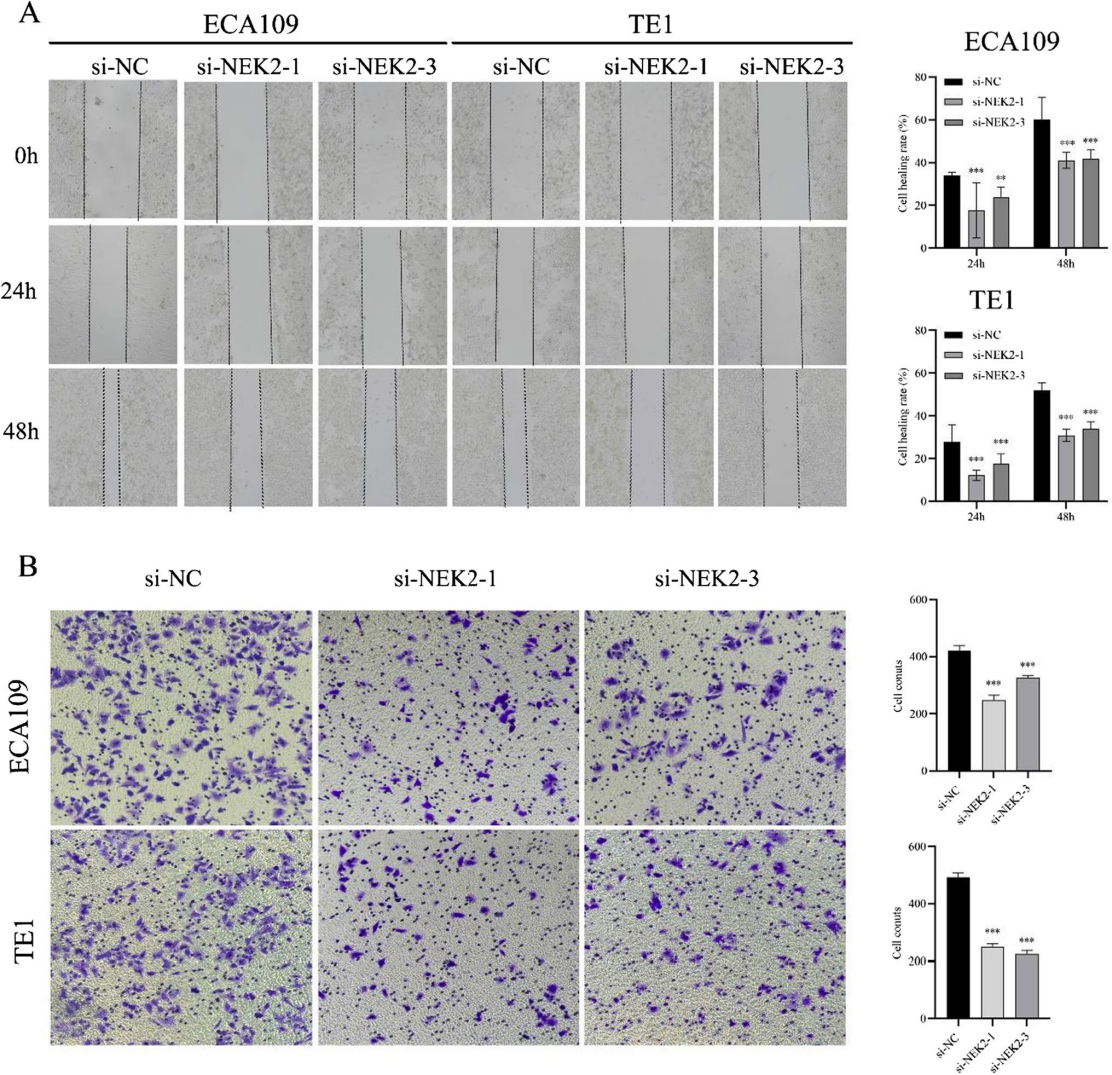
NEK2 enhanced the migration and invasion of ESCC cells. **A** The migratory capability was evaluated by wound-healing assay. **B** Transwell assay was used to detect the effect of NEK2 knockdown on invasion of ESCC cells. *ESCC* esophageal squamous cell carcinoma. *P < 0.05, **P < 0.005, ***P < 0.001

We conducted the wound-healing and transwell assays to investigate the migration and invasion roles of NEK2 in ESCC cells. For the wound-healing assay, the results indicated that the ESCC cells with knockdown of NEK2 exhibited a weak wound-healing abilities than the si-NC group (all P < 0.05, Fig. 6A). Additionally, we found that in comparison with the si-NC group cells, the invasion ability of ECA109 and TE1 cells with NEK2 knockdown group was significantly lower (all P < 0.05, Fig. 6B). Based on above results, we inferred that NEK2 can enhance migration and invasion ability of ESCC cells.

### NEK2 triggered epithelial–mesenchymal transition (EMT) via the Wnt/β-catenin signaling

**Fig. 7:**
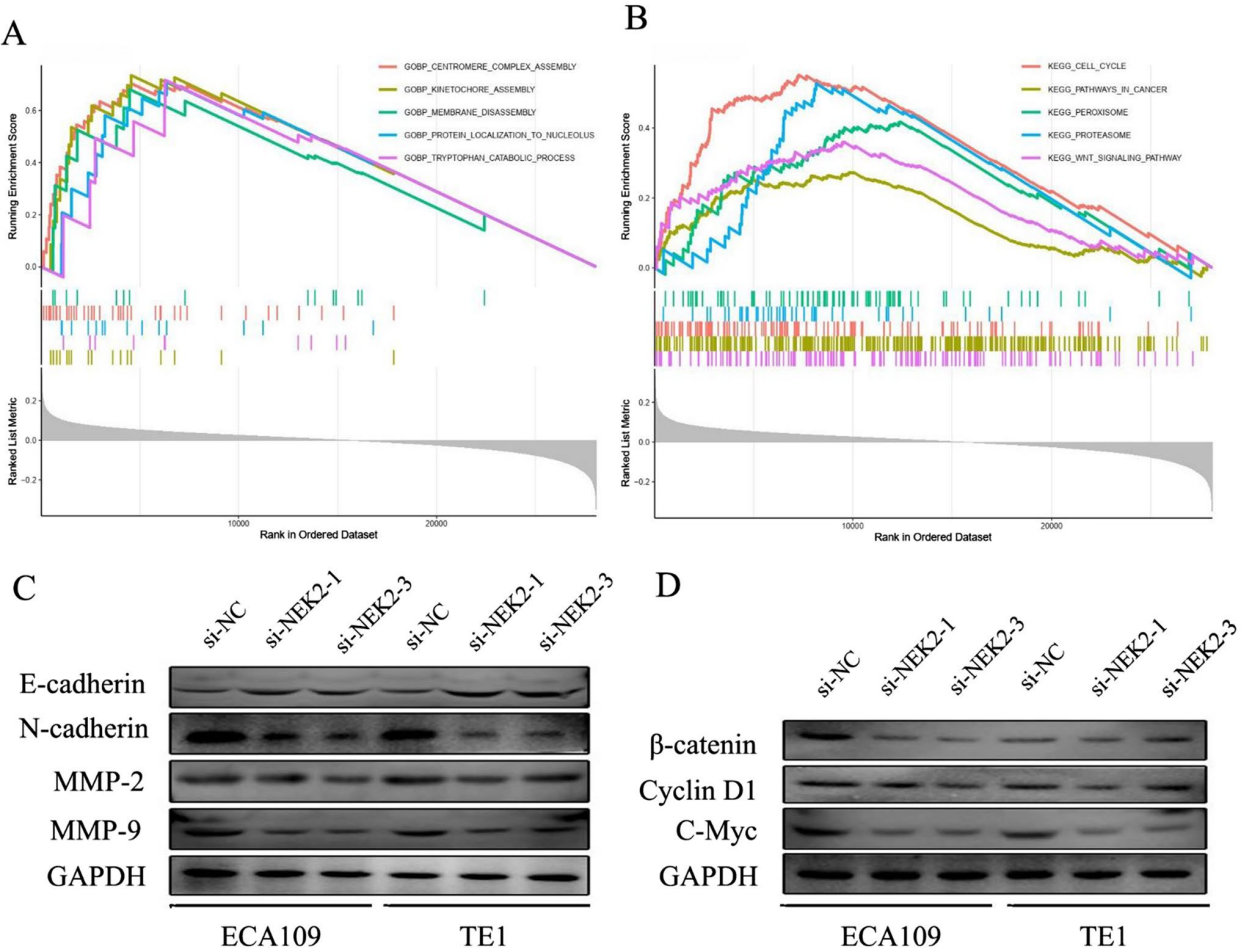
Wnt/β-catenin signaling pathway mediated NEK2-induced EMT of ESCC cells. **A**, **B** The GO analysis of biological processes and KEGG pathways by GSEA. **C** Western blot analysis of E-cadherin, N-cadherin, MMP-2, and MMP-9 in ECA109 and TE1 cell lines. **D** Western blot analysis of β-catenin, cyclin D1, and c-Myc in ECA109 and TE1 cell lines. *ESCC* esophageal squamous cell carcinoma. *P < 0.05, **P < 0.005, ***P < 0.001

**Fig. 8:**
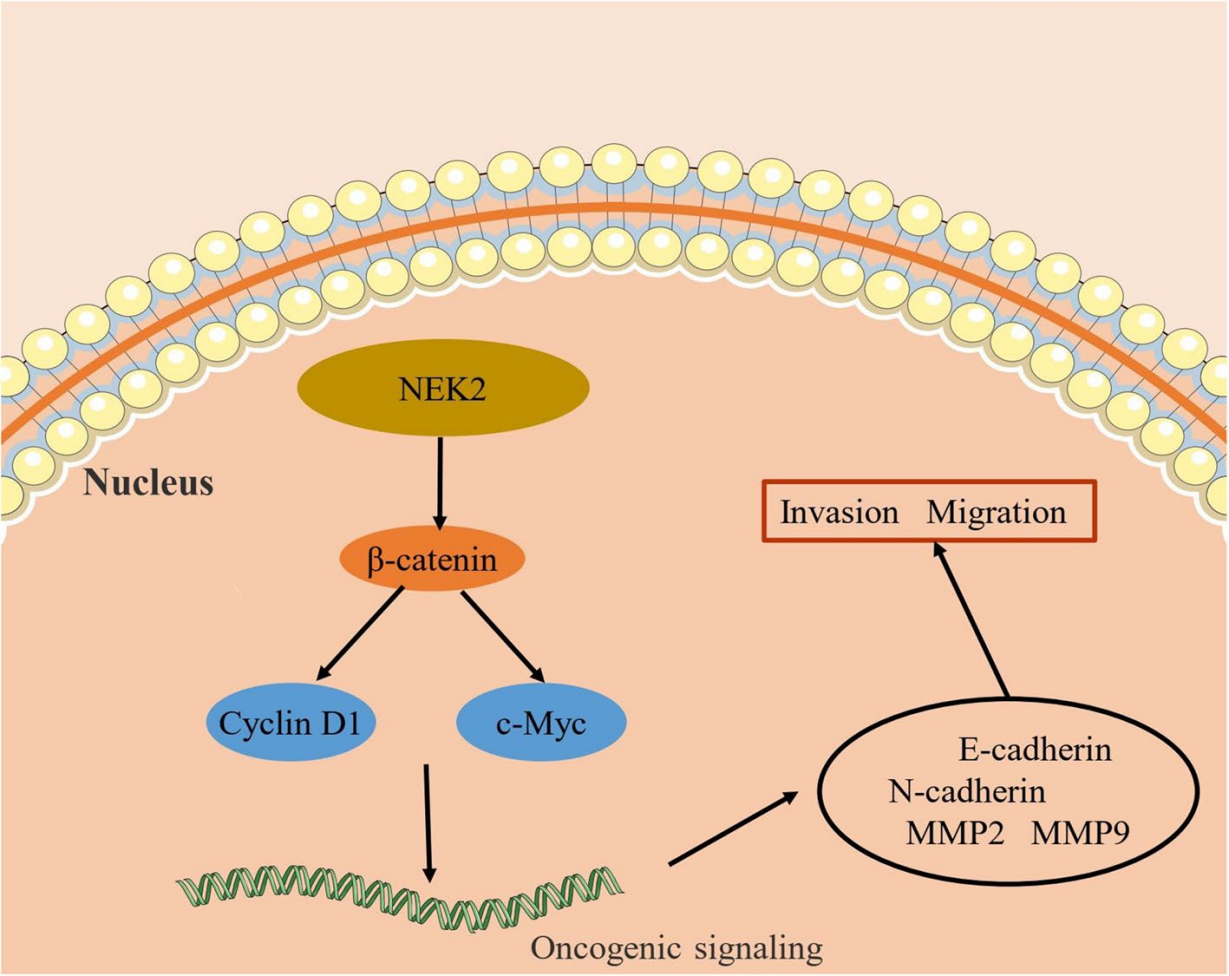
NEK2 promotes the ESCC cells proliferation, migration and invasion by activating Wnt/β-catenin signaling pathway

EMT is the prominent hallmark associated with cell movement, and the EMT progression often results in distant metastasis of ESCC cells [29]. To further explore the potential mechanism by which NEK2 affects ESCC EMT, we performed GSEA based on the NEK2 expression. The results showed that the Wnt signaling pathway and cancer signaling pathway were significantly enriched in the NEK2 high expression group (Fig. 7A, B). We speculated whether Wnt signaling pathway may be regulated by NEK2-mediated alterations in the EMT of ESCC cells. Subsequently, we analyzed the WB assay to validate this conjecture. In the NEK2 knockdown cells, N-cadherin, MMP-2 and MMP-9 expression was found to be decreased, whereas the expression of E-cadherin was the increased (Fig. 7C). We next examined the expression level of the Wnt/β-catenin pathway-related proteins. The results demonstrated that the expression of β-catenin, cyclin D1, and c-Myc decreased after knockdown of NEK2 (Fig. 7D). Above findings indicated that NEK2 promotes the EMT by activating Wnt/β-catenin signaling pathway (Fig. 8).

## Discussion

ESCC is a common digestive system tumor, and molecular targeted therapy for ESCC have been the research hot topics. With growing researches into molecular alterations that could contribute to tumorigenesis, the recognition of underlying molecules taking part in tumorigenesis processes is crucial and may find reliable targets for tumor-specific treatments. Currently, some tumorigenesis-associated molecular proteins had been applied to evaluate the diagnosis of ESCC in the clinical setting [30, 31]. Nevertheless, these molecular proteins did not meet the expectations, and their sensitivity and specificity are not ideal [32]. Therefore, the treatment efficacy and survival outcomes for ESCC patients could be improved by the identification of tumor-specific markers that can be applied to guide the personalize therapy in the age of precision medicine .

Centrosome replication can contribute to the tumorigenesis and the NIMA-related kinases regulating centrosome in the tumorigenesis process are often controlled by specific signal modulation [33]. NEK2, a member of the NIMA-related kinases family, located in the centrosome, is essential for spindle formation in cell cycle regulated process [34]. Dysregulation of this gene could result in the multiple signal regulation disorder, which eventually cause tumorigenesis. NEK2 has been demonstrated to inhibit gastric cancer cells proliferation, migration and tumor growth by activating the KDM5B/H3K4me3 signaling pathway [35]. Inhibiting NEK2 kinase activity can imped mitotic progression and result in apoptosis in colorectal cancer cells [36]. Feng et al. reported that NEK2 deficiency inhibit tumor proliferation and enhance the drug sensitivity against multiple myeloma, and NEK2 could act as a target of precision therapy in multiple myeloma with p53 abnormalities [37]. In addition, Zhang et al. found that NEK2 promote cervical cancer cells proliferation by decreasing Ser127-phosphorylation of the YAP protein. More importantly, NEK2 could inactivate the Hippo signaling pathway, resulting in growth-promoting genes high expression in cervical cancer [38]. In present study, we first showed that NEK2 was highly expressed in ESCC and can promote the tumor cells metastasis by EMT. Meanwhile, NEK2 knockdown suppressed EMT through regulating the Snail signaling pathway, which remains unclear yet. After performing deep mining of TCGA-ESCC and GEO datasets, we identified a profile of genetic variation of NEK2 in ESCC based on the mRNA expression data. Our results found that the expression level of NEK2 alters significantly changes in ESCC cell lines and tissues, which has been confirmed through qRT-PCR and western blotting. qRT-PCR and western blotting results showed that the NEK2 mRNA and protein levels in ECA109 and TE1 cell lines were significantly higher expression than in HEEC. These findings proved that NEK2 is a pro-oncogene from different aspects, which is consistent with the previous literature reports [39]. Importantly, the high NEK2 expression was significantly correlated with worse survival. Sequentially, we conducted cells assays to analyze the biological functions of NEK2 in ESCC. Our data revealed that knockdown NEK2 suppressed the ESCC cells proliferation, migration and invasion abilities. Taken together, these findings demonstrated the that NEK2 exerts a vital role for tumorigenesis and development of ESCC.

In addition to the above biological functions, NEK2 involved the regulation of EMT process. The epithelial cells get rid of cell type constraints by altering the structural proteins that maintain cell stability, resulting in the tumor cells migration and invasion [40, 41]. Tumor cells metastasis is a highly regulated process and a cascade of dynamic events involving many factors, and EMT activation is considered to be an important part of this process [42]. Zhang et al. found that EMT was the risk factor of ESCC, and can promote the ESCC metastasis [43]. We next conducted cells assay to analyze whether NEK2 could regulate the EMT process in ESCC. Our data revealed that NEK2 knockdown could upregulate the expression of E-cadherin and downregulate the mesenchymal markers (N-cadherin), MMP-2 and MMP-9 expression. These results further confirmed our hypothesis.

We next identify the NEK2-related the regulatory pathway through GSEA. Interestingly, the results revealed the Wnt signal was closely associated with NEK2 expression. Previous report has demonstrated that Wnt/β-catenin signaling pathway generate the important effect on EMT in ESCC [44]. Except for EMT biological behavior, the Wnt/β-catenin signaling pathway is also significantly related to tumor cell proliferation. β-catenin is the core member of the Wnt signaling pathway, which participates the tumor invasion and associates with EMT [45, 46]. β-Catenin can translocate to the nucleus and regulates the expression of Wnt target molecules such as cyclin D1 and c-myc [47]. These target molecules stimulate tumor proliferation, invasion, and migration [48]. Consequently, the study on NEK2 and whether it targets the Wnt/β-catenin signaling pathway may be important for the precise management of ESCC patients. In present study, we discovered that the expression of N-cadherin, MMP-2 and MMP-9 were downregulated by NEK2 Knockdown, and NEK2 knockdown decreased the expression of β-catenin, c-Myc and cyclin D1. These findings indicate that NEK2 can activate EMT by the Wnt/β-catenin signaling pathway in ESCC.

Despite obtaining the gratifying findings in present study, some limitations still existed. First, we explored the biology functions of NEK2 in ESCC cells proliferation, migration and invasion. Nevertheless, vivo assays were not been performed, which should be focused in future research. Second, the prognostic value of NEK2 in ESCC was identified in multiple public databases. A prospective survival analysis of clinical patients should be performed for verify. Finally, the concrete molecular regulatory mechanisms between the NEK2 and Wnt/β-catenin pathway should systematically be elucidated in vivo and in vitro.

## Conclusion

Our study demonstrated that NEK2 was upregulated in ESCC cell lines. High expression of NEK2 was associated with poor survival. Importantly, NEK2 enhanced ESCC cells proliferation, migration, invasion abilities and EMT, which was regulated by Wnt/β-catenin signaling pathway. These findings suggested that the NEK2 could serve as potential therapeutic target for ESCC patients.

## Data Availability

The datasets generated during and/or analyzed during the current study are available from the corresponding author on reasonable request.
